# Penile Calciphylaxis in End Stage Renal Disease

**DOI:** 10.1155/2013/968916

**Published:** 2013-06-06

**Authors:** Vincenzo Barbera, Luca Di Lullo, Antonio Gorini, Giovanni Otranto, Fulvio Floccari, Moreno Malaguti, Alberto Santoboni

**Affiliations:** ^1^Department of Nephrology and Dialysis, Colleferro County Hospital, Piazza Aldo Moro, Colleferro, 1-00034 Rome, Italy; ^2^Department of Nephrology and Dialysis, Civitavecchia County Hospital, Largo Donatori di Sangue, Civitavecchia, 1-00053 Rome, Italy

## Abstract

Calciphylaxis, better described as “Calcific uremic arteriolopathy” (CUA), involves about 1–4% of hemodialysis patients all around the world with high mortality rates. We describe a rare clinical case of CUA in peritoneal dialysis patient associated with urological disease. Penile calciphylaxis represents rare clinical complication, and an early diagnosis and multidisciplinary approach are requested. Pathogenesis is still unclear, and therapeutic approaches need more long-term clinical trials to test their efficacy and safety.

## 1. Case Report

We describe a case report of a 50-year- (at his first referring in our nephrological unit) Caucasian man with clinical history of recurrent upper respiratory tract infections since the age of eight, consequent rheumatic disease, and mitral valve involvement. At the age of 20, the patient underwent surgery therapy for mitral commissural annuloplasty. The following year was carried clinical diagnosis of chronic glomerulonephritis (renal biopsy not performed) together with arterial hypertension development and progression towards chronic kidney disease. Ten years later (at the age of 31), the patient underwent on surgery for mitral graft implantation. Anticoagulant (with dicumarolic agents) therapy was started. Renal function worsened in the next 12 years, and patient started peritoneal dialysis by CAPD (four daily exchanges with 2000 mL of 1.36% solution). At this time (June 1996), total calcemia was 10.1 mg/dL, phosphatemia 3.8 mg/dL, Ca-P product 38.4, and iPTH < 300 pg/mL; patient's residual diuresis was about 1000 mL/die. Blood pressure was poorly controlled, while phosphate binding therapy (with aluminium hydroxide and calcium carbonate) was started. Three months later, CKD-MBD pattern got worsened (total calcemia up to 12.8 mg/dL, serum phosphate up to 8.6 mg/dL, and Ca × P product up to 111). Eight months later (May 1997), iPTH raise up to 615 pg/mL; calcitriol therapy (0.5 mcg/die dosage) was started with normalization of calcium and phosphate serum levels. Two years later, despite patient's good therapeutic and dietetic compliance, iPTH raise up to 1200 pg/mL. To achieve both metabolic and dialytic parameters better control, APD-TIDAL peritoneal dialysis was started (June 1999) with fast decrease in iPTH, calcium, and phosphate levels.

In December 2000, iPTH raise up to 800 pg/mL again; calcitriol intravenous therapy was started with poor results.

In July 2001, due to recurrent pulmonary infective relapse, thoracoabdominal CT scan was performed. CT scan showed presence of right posterior basal nodular area, strictly close to parietal pleura with small lymph nodes on Barety lodge. In April 2002, patient developed an ulcerous lesion on glans' dorsoventral surface. Histology showed “…presence of multiple calcific areas on medial tunica, partially occluding arterial lumen, with associated endoluminal thrombosis. Osteoid apposition on pannicular arteries of dermal tissue. Soft tissues calcification are evident…”.

Few days later, patient was hospitalized for penile edema with ulcerogangrenous evolution. Penile ecocolor doppler investigation showed “Severe penile artery sclerosis with several calcific plaques and marked hyposphigmia.” 

**Figure 1:**
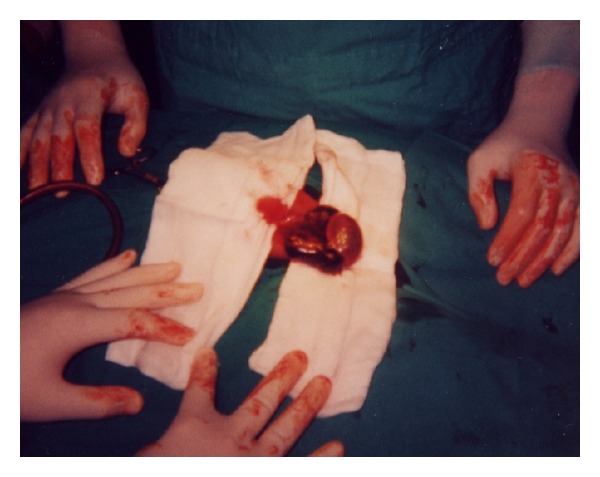
Patient's preparation.

**Figure 2:**
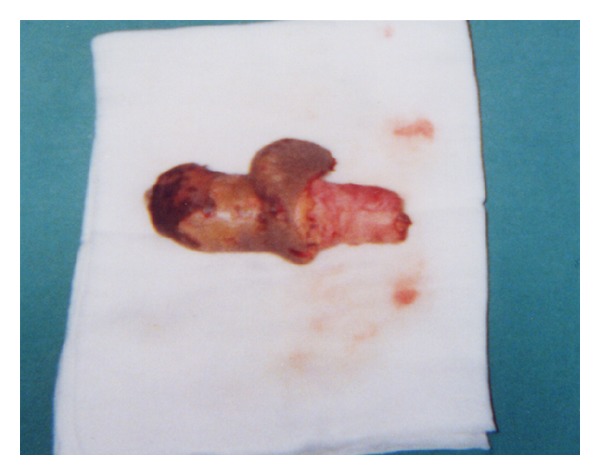
Penile ventral surface.

In April 2002, both for recurrent peritonitis and scheduled penectomy (performed on April 30th 2002) (Figures 1 and 2), patient was shifted to hemodialytic treatment.

In February 2003, patient was hospitalized for bowel subocclusion (stenotic lesion at proximal bowel); CT scan was diagnostic for multiple calcific areas on right posterior basal pulmonary side with right anterolateral pleural calcification and diffuse parenchymal micronodular calcific lesions; pretracheal lymphoadenopathy was also detected.

In March 2003, patient was hospitalized again for bowel occlusion recurrence: on explorative laparotomy, multiple gangrenous lesions on right colon were found (as a result of mesenteric arterial infarction). One week later, necrotic lesion on right foot second finger occurred. Patient's clinical condition worsened in the next months until he died in April 2003. 

## 2. Discussion

Calciphylaxis, better described as “Calcific uremic arteriolopathy” (CUA), is characterized by small and medium arterial medial tunica calcifications and involves about 1–4% of hemodialysis patients all around the world [1, 2] with high mortality rates [3].

Term “calciphylaxis” was initially postulated in middle 19th century when Virchow described unlikely association between chronic kidney disease and soft tissues calcifications. Selye [4] referred those peculiar lesions to an atopic disorder.

Main clinical finding is represented by heavy pain, while ischemic cutaneous lesions show wide spectrum of involvement from small areas of livedo reticularis and single plaques to ulcerous and necrotizing lesions development [5]; necrotic areas can infect themselves leading to sepsis and increased mortality rates in these patients.

Body areas mainly involved are represented by upper and lower limbs, buttocks, thighs, and trunk; proximal involvement is usually linked with worse prognosis [6]; visceral involvement is more common than expected [7].

Our patient showed CT scan pulmonary nodular images, but we cannot surely be ascribed to visceral calciphylaxis because of periodic bronchopneumonic infective relapse that has led to microcalcific outcomes. Spleen involvement itself could be ascribed to long lasting peritoneal dialysis (six years) with several peritonitis events and resulting peritoneal adhesions “till mesenteric artery infarction.”

Pathogenetic pathways of CUA are still partially unclear. Since CUA is mainly diagnosed in hemodialysis patients, uremic status could be the main risk factor to develop calciphylaxis in end stage kidney disease patients. On the other hand, bone mineral imbalance (such as raising in phosphate and iPTH serum levels) could play a crucial role in pathophysiological pathways leading to vascular calcifications in CUA.

There is no overall agreement on parathyroidectomy, although it could relieve pain and expedite skin wounds recovery [8], especially in cinacalcet nonresponders patients.

Close control of phosphatemia with phosphate-binding agents (such as lanthanum and sevelamer carbonate) is crucial to prevent clinical development of CUA [9].

Development of calciphylaxis is often supported by antithrombotic agents therapy [10, 11]; Gla protein activity is strictly dependent on vitamin K oxidative carboxylation blocked by oral antithrombotic therapy with warfarin. Sodium thiosulfate has provided good result in treating calciphylaxis cutaneous wounds. Its role as a rather potent antioxidant has uniquely been associated with a prompt decrease in pain, and its slower chelating properties are associated with regression of subcutaneous calcifications [12]. Hyperbaric oxygen therapy, increasing oxygen delivery to tissues, seems to promote cutaneous lesions healing promoting neoangiogenesis, fibroblastic growth, and collagen expression.

New therapeutic devices are represented by *lucilia Sericata* larvae both secreting proteolytic enzymes (able to clear away necrotic tissue from skin lesions) and phenylacetic acid with powerful antibacterial activity. Etidronate and pamidronate employment is still to be assessed.

## 3. Conclusions

Penile calciphylaxis represents rare clinical complication in chronic kidney disease systemic CUA. Because of high mortality risk, early diagnosis and multidisciplinary approach (nephrologist, urologist, vascular surgeon, and dermatologist) are requested. 

Pathogenesis is still unclear, and therapeutic approaches need more long-term clinical trials to test their efficacy and safety.
